# Clinical Features and Risk Factors of ICU Admission for COVID-19 Patients with Diabetes

**DOI:** 10.1155/2020/5237840

**Published:** 2020-12-11

**Authors:** Ming Lei, Kashuai Lin, Yaoqiu Pi, Xiaomei Huang, Lixin Fan, Jun Huang, Riguang Liu, Lin Liu, Xinning Shao, Kaiyuan Hu, Liuping Yang, Shuguang Qin, Feng He

**Affiliations:** ^1^Guangzhou Eighth People's Hospital, Guangzhou Medical University, Guangzhou, China; ^2^Guangzhou First People's Hospital, School of Medicine, South China University of Technology, Guangzhou, Guangdong, China

## Abstract

**Introduction:**

Previous studies of coronavirus disease 2019 (COVID-19) have focused on the general population. However, diabetes (DM) as one of the most common comorbidities is rarely studied in detail. This study is aimed at describing clinical characteristics and determining risk factors of ICU admission for COVID-19 patients with DM.

**Methods:**

Data were extracted from 288 adult patients with laboratory-confirmed COVID-19 from Guangzhou Eighth People's Hospital. Demographic characteristics, laboratory results, radiographic findings, complications, and treatments were collected and compared between DM and non-DM groups. Binary logistic regression was used to identify the risk factors associated with ICU admission for COVID-19 patients with DM or non-DM.

**Results:**

COVID-19 patients with DM showed as older ages, higher levels of C-reactive protein (CRP), myoglobin, alanine transaminase (ALT), and aspartate transaminase (AST). They were also more prone to transfer to the intensive care unit (ICU) for treatment. Multiple regression analysis showed that the following were the independent risk factors for COVID-19 patients with DM that received ICU admission: each 1-year increase in age (odds ratio (OR), 1.07; 95% CI, 1.02-1.13; *P* = 0.007), respiratory rate over 24 times per minute (OR, 5.22; 95% CI, 2.26-16.58; *P* = 0.016), HbA1c greater than 7% (OR, 4.58; 95% CI, 1.82-10.55; *P* = 0.012), and AST higher than 40 U/L (OR, 2.96; 95% CI, 1.58-8.85; *P* = 0.022). In addition, each 1-year increase in age (OR, 1.05; 95% CI, 1.01-1.10; *P* = 0.006), diarrhea (OR, 4.62; 95% CI, 2.01-9.36; *P* = 0.022), respiratory rate over 24 times per minute (OR, 5.13; 95% CI, 1.18-16.82; *P* = 0.035), CRP greater than 10 mg/L (OR, 5.19; 95% CI, 1.37-13.25, *P* = 0.009), and TnI higher than 0.03 *μ*g/L (OR, 6.48; 95% CI, 1.17-21.38; *P* = 0.036) were risk factors for ICU admission of COVID-19 patients with non-DM.

**Conclusions:**

The older age, respiratory rate over 24 times per minute, HbA1c greater than 7%, and AST higher than 40 U/L were risk factors of ICU admission for COVID-19 patients with diabetes. Investigating and monitoring these factors could assist in the risk stratification of COVID-19 patients with DM at an early stage.

## 1. Introduction

Coronavirus disease 2019 (COVID-19) is a newly discovered infectious disease caused by Severe Acute Respiratory Syndrome Coronavirus 2 (SARS-CoV-2) [1, 2], which is raging all over the world at an unprecedented rate. Until July 26, 2020, more than 16 million cases have been reported in 215 countries and regions, with 652,739 deaths [3]. Given the terrible spread of COVID-19 and its substantial morbidity and mortality, the global health system has been overwhelmed and even affected the development of the global economy.

Diabetes is one of the most common comorbidities in patients hospitalized with COVID-19, since most previous large studies have reported that diabetes is present in 19-34% of this patient population [4, 5]. Moreover, some studies have found that patients with chronic diseases such as diabetes are more severe and have worse prognosis, including to be admitted to the intensive care unit (ICU) [6–8]. In addition, a recent meta-analysis of 1382 diabetic patients showed that COVID-19 patients with diabetes had a higher risk of ICU admission [9]. However, the risk factors for ICU admission of COVID-19 patients with diabetes are currently unclear. Meanwhile, glycemic control in diabetics appears to be an important prognostic factor for any form of infection [10–12]. Although some studies have shown that hyperglycemia is a risk factor for the poor prognosis of COVID-19 with DM, and even associated with higher mortality in diabetes [13–15], there is a lack of information on the relationship between blood glucose control and the prognosis in COVID-19 patients with DM. In order to explore the influence of hyperglycemia on the prognosis of COVID-19 patients with diabetes, it is necessary to conduct further investigations. Therefore, the present study is aimed at describing clinical characteristics and identifying risk factors for the ICU admission of COVID-19 patients with DM.

## 2. Methods

### 2.1. Study Design and Participants

This single-center, retrospective cohort study was conducted at Guangzhou Eighth People's Hospital (Guangzhou, China), which is a designed hospital for patients with COVID-19. 288 laboratory-confirmed patients hospitalized from January 15, 2020, to March 10, 2020, were enrolled in our study, including 24 patients combined with DM.

This study was approved by the Ethics Committee of Guangzhou Eighth People's Hospital, and informed consent was obtained from all patients enrolled.

### 2.2. Data Collection and Definition

Information extracted from clinical electronic records includes clinical features, signs and symptoms, comorbid conditions, chest computed tomography (CT), and laboratory examination results, as well as the patient's treatment and outcomes. The clinical and laboratory parameters were measured when the patients were admitted to the hospital.

According to the Chinese diagnosis and treatment guideline for COVID-19 (trial version 7.0) [16], severe cases were defined as including one of the following criteria: (1) respiratory rate > 30/min, (2) oxygen saturation ≤ 93%, and (3) PaO_2_/FiO_2_ ≤ 300 mmHg. Severe patients who need high-flow nasal intubation or higher levels of oxygen support to correct hypoxemia, or multiple organ dysfunction, are admitted to the ICU. Diabetes was ascertained through a diabetes diagnosis in medical records or a self-reported diagnosis confirmed by medical records reviewed by endocrinologists. Diabetes was defined according to the World Health Organization diagnostic criteria: fasting plasma glucose ≥ 7.0 mmol/L (≥126 mg/dL) or 2 h plasma glucose ≥ 11.1 mmol/L (≥200 mg/dL). CVD was defined as the clinical diagnosis of coronary heart disease, cerebrovascular disease, peripheral arterial disease, rheumatic or congenital heart diseases, or venous thromboembolism [17]. Acute Respiratory Distress Syndrome (ARDS) was defined according to WHO's guidance for COVID-19 [18]. Chronic kidney disease was defined as either eGFR of <60 mL/min/1.73 m^2^ according to the KDIGO clinical practice guidelines [19]. The reference ranges of all laboratory inspection indicators were measured in the laboratory of Guangzhou Eighth People's Hospital.

### 2.3. Statistical Analysis

We represented continuous variables as median and interquartile range (IQR) and categorical variables as frequency (*N*) and percentage (%). We assessed differences between diabetic patients and nondiabetic patients using a two-sample *t* test or the Mann-Whitney *U* test depending on parametric or nonparametric data for continuous variables and the *χ*^2^ test or Fisher's exact test for categorical variables. Binary logistic regression was used to explore risk factors associated with ICU admission for COVID-19 patients with DM or non-DM. The multivariable logistic regression model was constructed using all variables in univariate logistic regression analysis.

A *P* value of less than 0.05 was considered statistically significant. The SPSS 22.0 software was used for all analyses.

## 3. Results

From January 15, 2020, to March 10, 2020, clinical data of 292 laboratory-confirmed COVID-19 patients were collected from Guangzhou Eighth People's Hospital. After excluding 4 pediatric patients, we finally included 288 adult patients into the cohort, including 24 patients combined with DM. The median age was 48.5 years (IQR, 34.3-62.0), of which women accounted for 54.5% (Table 1). Compared with non-DM (47.0 (IQR, 33.0-61.0)), the DM group was significantly older (62.5 (IQR, 55.50-64.75); Table 1). Cardiovascular disease was the most common complication in patients with DM (16 (66.7%); Table 1). Fever (201 (69.8%)) and cough (163 (56.6%)) were the most common symptoms at the onset of illness for all patients, followed by sore throat (67 (23.3%)) and sputum production (58 (20.1%)). Compared to non-DM patients (14 (5.3%)), the respiratory rate of COVID-19 patients with DM (5 (20.8%)) was more likely to exceed 24 times per minute (Table 1), meaning shortness of breath.

**Figure 1:**
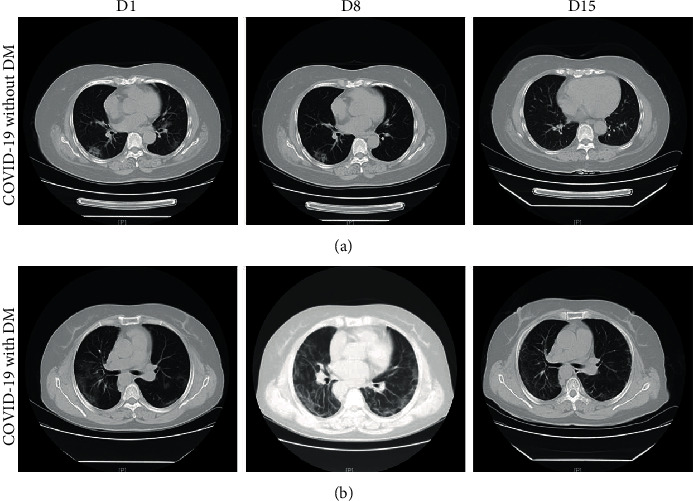
Representative chest computed tomography (CT) images of COVID-19 pneumonia in a nondiabetes mellitus (DM) case and a DM case. (a) A 54-year-old woman with COVID-19, but not DM: chest CT images showed ground-glass opacity (GGO) and patchy consolidation with peripheral and subpleural distribution, which had been absorbed at 15 days after hospitalization with treatment. (b) A 58-year-old woman with both COVID-19 and DM: chest CT images showed bilateral multiple lobules and subpleural diffuse consolidation. Chest CT showed that there was still a small amount of bilateral ground-glass opacity at 15 days after hospitalization with treatment.

Laboratory data showed leucopenia (white blood cell count < 4 × 10^9^/L) occurred in 62 (21.5%) patients and lymphopenia (lymphocyte count < 1.1 × 10^9^/L) occurred in 91 (31.6%) patients, with no significant difference between the non-DM group and the DM group (Table 2). Compared to COVID-19 patients without DM, the levels of C-reactive protein (CRP), myoglobin, alanine transaminase (ALT), and aspartate aminotransferase (AST) were significantly increased in DM patients (Table 1). As for the results of chest CT images, the most common pattern was bilateral pulmonary infiltration (241 (83.7%)), of which 22 (91.7%) were diabetic patients (Table 1). Notably, COVID-19 patients without DM had relatively favorable manifestations of chest CT images, including bilateral multifocal ground-glass opacities and patchy consolidations (Figure 1(a)). In contrast, COVID-19 patients with DM showed a rapid and worsening radiographic progression, bilateral multiple lobules, and subpleural diffuse consolidation that were seen on CT images (Figure 1(b)).

More respiratory support therapy, continuous positive airway pressure (CPAP), and extracorporeal membrane oxygenation (ECMO) were more commonly used in DM patients (Table 2). We noticed that, despite having received more aggressive treatment against COVID-19 combined with DM, the diabetic group was more likely to develop into severe or critically severe cases and showed higher proportion of ICU admission, compared to the nondiabetic group (5 (20.8%) vs. 22 (8.3%); Table 2).

In univariate logistic regression analysis, we found that higher odds of ICU admission were related to older age, respiratory rate over 24 breaths per minute, and increased levels of HbA1c, ALT, and AST in patients with DM (Table 3). In the multivariable logistic regression analysis, we found that each 1-year increase in age (OR, 1.07; 95% CI, 1.02-1.13; *P* = 0.007), the respiratory rate over 24 breaths per minute (OR, 5.22; 95% CI, 2.26-16.58; *P* = 0.016), HbA1c greater than 7% (OR, 4.58; 95% CI, 1.82-10.55; *P* = 0.012), and AST higher than 40 U/L (OR, 2.96; 95% CI, 1.58-8.85; *P* = 0.022) were independent risk factors for the ICU admission of COVID-19 patients with DM (Table 3). Besides as shown in Table 4, risk factors for ICU admission of COVID-19 patients with non-DM were each 1-year increase in age (OR, 1.05; 95% CI, 1.01-1.10; *P* = 0.006), diarrhea (OR, 4.62; 95% CI, 2.01-9.36; *P* = 0.022), respiratory rate over 24 times per minute (OR, 5.13; 95% CI, 1.18-16.82; *P* = 0.035), CRP greater than 10 mg/L (OR, 5.19; 95% CI, 1.37-13.25, *P* = 0.009), and TnI higher than 0.03 *μ*g/L (OR, 6.48; 95% CI, 1.17-21.38; *P* = 0.036).

## 4. Discussion

We reported a retrospective cohort study of 288 adult hospitalized patients with COVID-19, including 24 patients with DM. Compared with the non-DM patients, patients with DM were older and had higher levels of CRP, AST, and ALT, more often combined with CVD. These patients were also more likely to develop into severe or critically severe cases and to be admitted to the ICU. Particularly, multivariable regression revealed that older age, respiratory rate over 24 times per minute, HbA1c greater than 7%, and AST higher than 40 U/L were associated with increasing odds of ICU admission in COVID-19 patients with DM.

The median age of COVID-19 patients with DM was significantly older than that of patients without DM (Table 1). According to previous reports, older patients were at higher risk for severe COVID-19 and eventually receive ICU admission and even death [8]. In our study cohort, older age was also one of the risk factors of ICU admission in COVID-19 patients with DM or non-DM. Although the pathophysiological mechanisms are still not understood, it may be explained by the dysfunction of the immune system with aging [20, 21]. In addition, a study reported that older age was related to defects in T-cell and B-cell function and excess inflammation markers, which could be detrimental to the control of viremia and inflammation, aggravating morbidity and mortality in older patients [5]. Thus, COVID-19 patients with DM were prone to developing severe cases and even to be admitted to the ICU that may be related to age-dependent immune defects and dysregulated proinflammatory response. Moreover, our results show that patients with DM have a higher CRP level. The increase in CRP may be related to a severe inflammatory cascade, which could induce the cytokine storm and lead to multiple organ dysfunction [2, 21]. Furthermore, abnormal delayed-type hypersensitivity reaction [22] and complement activation dysfunction [23] have also been described in patients with diabetes. Therefore, it is reasonable to speculate that elderly patients with DM may lead to inadequate control of viral replication and longer proinflammatory response, potentially leading to poor prognosis, including ICU admission.

In our cohort, compared with non-DM patients, patients with DM had a higher level of AST. AST higher than 40 U/L was associated with increasing odds of ICU admission of COVID-19 patients with DM. Recently, a study published in the Lancet found that patients with severe COVID-19 seemed to have more liver dysfunction [2]. Consistent with this finding, our result showed that liver injury was an important feature of disease progression in patients with DM. While the mechanism of liver injury is not fully understood, this may be the result of the virus directly interacting with liver cells or synergistically with the immune response [24, 25]. It was suggested that routine monitoring of liver function in COVID-19 patients during hospitalization would be important.

Our study also found respiratory rate over 24 breaths per minute with adverse outcomes of ICU admission in COVID-19 patients with DM. There was a significant difference in the incidence of shortness of breath between diabetic and nondiabetic patients (*P* = 0.003). In patients with diabetes, a variety of pulmonary dysfunction including significant reduction in forced vital capacity and forced expiratory volume in one second has been reported, which may account for the propensity of poor outcomes in patients with COVID-19 and DM [26]. Previous studies reported that the association between diabetes and lung dysfunction may be partly explained by systemic inflammation [27, 28]. Indeed, diabetes is widely considered to be a chronic, low-grade inflammatory disease [29]. Meanwhile, SARS-CoV-2 can directly interact with the islets, thus aggravating the systemic inflammatory state of diabetes [30]. Moreover, studies showed that SARS-CoV-2 invasion of the lungs through angiotensin-converting enzyme 2 (ACE2) results in severe respiratory dysfunction and hypoxemia [31]. Combined with the above effects, it is suggested that patients with diabetes are more likely to develop pulmonary function deterioration and are more likely to develop into severe or critical cases and be sent to the ICU.

It is very noteworthy that HbA1c greater than 7% was a risk factor for ICU admission in COVID-19 patients with DM. Previous studies have reported that patients with poor HbA1c control were more susceptible to infections and exhibited worse prognosis and even death compared to the patients with well HbA1c control [32]. Moreover, recent studies have shown that hyperglycemia was associated with higher odds of disease worsening of COVID-19 patients with DM [13–15]. This may be related to the following mechanisms: chronic hyperglycemia was thought to downregulate ACE-2 expression, making cells susceptible to the damage of SARS-CoV-2 [33]. Meanwhile, SARS-CoV-2 can directly damage the islets of *β*-cells, and blood glucose is even more out of control [34]. In addition, hyperglycemia is not conducive to the control of viremia and inflammation, which aggravates the patient's condition [13]. Furthermore, hyperglycemia has been shown to damage immune function, especially the innate immune system, and increase inflammatory cytokines, such as interleukin-6 [14, 35]. Our study found that in COVID-19 patients with DM, those with elevated HbA1c had a higher risk of disease deterioration and then to be admitted to the ICU. This suggests that glycemic control is strongly associated with the severity of COVID-19 patients with DM.

There are several limitations that should be noted. First, due to the retrospective study design, we were unable to obtain the dynamic changes and the concentration data of important inflammatory cytokines (such as interleukin-6, MCP1, IL-1*β*, and IFN-*γ*). Thus, we have no direct evidence to determine whether the cytokine storm happened in these COVID-19 patients. Second, as a single-center study, our conclusion may be limited by a small sample size and selection bias. Third, not all patients have tested HbA1c levels. Therefore, their role in blood glucose control might be underestimated. The data of this study is only a preliminary evaluation of the clinical characteristics of COVID-19 patients with DM and, based on the existing data, to a preliminary exploration of the risk factors associated with ICU admission for COVID-19 patients with DM or non-DM. Further researches are still needed.

In summary, compared to COVID-19 patients without DM, our study found that COVID-19 patients with diabetes were more likely to be admitted to the ICU. Furthermore, we determined that older age, respiratory rate over 24 times per minute, HbA1c greater than 7%, and AST higher than 40 U/L were risk factors for ICU admission of COVID-19 patients with DM. Investigating and monitoring these factors could assist in the risk stratification of COVID-19 patients with DM, so that timely and aggressive interventions can be implemented at an early stage. It would also provide significant experience and references for global antiepidemic work in patients with DM.

## Figures and Tables

**Table 1:** Clinical features of COVID-19 patients with non-DM or DM.

Parameters	Total (*n* = 288)	Non-DM (*n* = 264)	DM (*n* = 24)	*P* value
*Clinical characteristics*				
Age, median (IOR), years	48.5 (34.3-62.0)	47.0 (33.0-61.0)	62.5 (55.50-64.75)	<0.0001
Age groups (years)				
≤30	44 (15.3%)	44 (16.7%)	0 (0.0%)	<0.0001
30-45	87 (30.2%)	85 (32.2%)	2 (8.3%)	
45-65	116 (40.3%)	97 (36.7%)	19 (79.2%)	
>65	41 (14.2%)	38 (14.4%)	3 (12.5%)	
Female, *n* (%)	157 (54.5%)	143 (54.2%)	14 (58.3%)	0.695
Diabetes duration (years)	NA	NA	10.8 (6.5-15.6)	NA
Types of diabetes				
Type 1	NA	NA	2 (8.3%)	NA
Type 2	NA	NA	22 (91.7%)	NA
*Comorbidities*				
Hypertension	84 (29.2%)	68 (25.8%)	16 (66.7%)	<0.0001
CVD	85 (29.5%)	69 (26.1%)	16 (66.7%)	<0.0001
COPD	5 (1.7%)	4 (1.5%)	1 (4.2%)	0.343
Chronic kidney disease	8 (2.8%)	6 (2.3%)	2 (8.3%)	0.084
Chronic liver disease	10 (3.5%)	8 (3.0%)	2 (8.3%)	0.174
*Signs and symptoms*				
Fever	201 (69.8%)	181 (68.6%)	20 (83.3%)	0.131
Chill	55 (19.1%)	52 (19.7%)	3 (12.5%)	0.390
Cough	163 (56.6%)	148 (56.1%)	15 (62.5%)	0.542
Expectoration	58 (20.1%)	52 (19.7%)	6 (25.0%)	0.535
Myalgia	35 (12.2%)	31 (11.7%)	4 (16.7%)	0.480
Fatigue	43 (14.9%)	40 (15.2%)	3 (12.5%)	0.727
Sore throat	67 (23.3%)	64 (24.2%)	3 (12.5%)	0.192
Headache	26 (9.0%)	24 (9.1%)	2 (8.3%)	0.901
Nausea or vomiting	29 (10.1%)	28 (10.6%)	1 (4.2%)	0.316
Diarrhea	11 (3.8%)	10 (3.8%)	1(4.2%)	0.926
Respiratory rate > 24 breaths per min	19 (6.6%)	14 (5.3%)	5 (20.8%)	0.003
Unilateral pneumonia	31 (10.8%)	30 (11.4%)	1 (4.2%)	0.274
Bilateral pneumonia	241 (83.7%)	219 (83.0%)	22 (91.7%)	0.269
*Laboratory variables*				
White blood cell (×10^9^/L)	5.20 (4.14-6.44)	5.20 (4.10-6.40)	5.39 (4.62-7.12)	0.840
White blood cell count, ×10^9^/L (No. (%))				
≤4	62 (21.5%)	58 (22.0%)	4 (16.7%)	0.825
4-10	216 (75.0%)	197 (74.6%)	19 (79.2%)	
>10	10 (3.5%)	9 (3.4%)	1 (4.2%)	
Lymphocyte count (×10^9^/L)	1.42 (1.04-1.96)	1.42 (1.04-1.95)	1.40 (1.07-2.16)	0.314
Lymphocyte count, ×10^9^/L (No. (%))				
<1.1	91 (31.6%)	83 (31.4%)	8 (33.3%)	0.848
Platelet count (×10^9^/L)	194.5 (158-247)	196.0 (160-247)	186.5 (142-228.5)	0.679
Hemoglobin (g/L)	135.5 (123-147)	136.0 (123-147)	133.5 (121-145.8)	0.656
Blood glucose (mmol/L)	4.97 (4.39-5.76)	4.56 (4.16-5.52)	7.95 (6.57-11.85)	0.001
HbA1c (%)	NA	NA	6.81 (5.91-7.95)	NA
HbA1c (No. (%))				
<7%	NA	NA	9 (37.5%)	NA
≥7%	NA	NA	15 (62.5%)	
Serum insulin (mIU/L)	NA	NA	20.68 (11.82-36.55)	NA
Procalcitonin (ng/mL)	0.13 (0.04-32.60)	0.13 (0.04-33.55)	0.097 (0.038-20.20)	0.733
C-reactive protein (mg/L)	9.00 (8.00-22.72)	9.00 (8.00-21.50)	21.15 (8.25-47.09)	0.039
C-reactive protein, mg/L (No. (%))				
≤10	175 (60.8%)	167 (63.3%)	8 (33.3%)	0.004
>10	113 (39.2%)	97 (36.7%)	16 (66.7%)	
D-dimer (*μ*g/L)	1100 (700-1700)	1090 (680-1700)	1190 (850-1770)	0.198
D-dimer (No. (%))				
<1000	125 (43.9%)	116 (44.3%)	9 (39.1%)	0.634
≥1000	160 (56.1%)	146 (55.7%)	14 (60.9%)	
Myoglobin (*μ*g/L)	15.00 (8.85-22.46)	14.75 (8.70-22.25)	19.00 (9.20-32.40)	0.003
Myoglobin, *μ*g/L (No. (%))				
<106	269 (97.1%)	247 (97.2%)	22 (95.7%)	0.662
≥106	8 (2.9%)	7 (2.8%)	1 (4.3%)	
Troponin I (*μ*g/L)	0.004 (0.001-0.009)	0.003 (0.001-0.009)	0.008 (0.002-0.017)	0.784
Creatine kinase (U/L)	52.00 (36.00-80.00)	52.00 (36.00-80.75)	51.00 (38.00-78.00)	0.351
Creatine kinase, U/L (No. (%))				
≤310	283 (98.6%)	261 (98.9%)	22 (95.7%)	0.208
>310	4 (1.4%)	3 (1.1%)	1 (4.3%)	
Creatinine (*μ*mol/L)	61.80 (50.25-76.58)	62.05 (50.05-76.68)	60.00 (51.63-73.95)	0.616
ALT (U/L)	22.45 (14.30-34.50)	22.40 (14.30-34.15)	24.25 (14.50-52.27)	0.889
ALT, U/L (No. (%))				
≤50	254 (88.2%)	237 (89.8%)	17 (70.8%)	0.006
>50	34 (11.8%)	27 (10.2%))	7 (29.2%)	
AST (U/L)	18.35(14.9-25.63)	18.20 (14.53-24.48)	24.75 (16.88-44.50)	<0.0001
AST, U/L (No. (%))				
≤40	256 (88.9%)	240 (90.9%)	16 (66.7%)	<0.0001
>40	32 (11.1%)	24 (9.1%)	8 (33.3%)	

Data are presented as median (interquartile range) or number (percent). Abbreviation: IQR: interquartile range; NA: not applicable; CVD: cardiovascular disease; COPD: chronic obstructive pulmonary disease; HbA1c: hemoglobin A1c; ALT: alanine transaminase; AST: aspartate aminotransferase.

**Table 2:** Treatments and outcomes of COVID-19 patients with non-DM or DM.

	Total (*n* = 288)	Non-DM (*n* = 264)	DM (*n* = 24)	*P* values
Treatments				
Antiviral	233 (80.9%)	215 (81.4%)	18 (75%)	0.442
Antibiotics	244 (84.7%)	223 (84.5%)	21 (87.5%)	0.693
Vasoactive drugs	5 (1.7%)	4 (1.5%)	1 (4.2%)	0.341
Glucocorticoid	21 (7.3%)	19 (7.2%)	2 (8.3%)	0.838
Oxygen inhalation				
None	88 (30.6%)	82 (31.1%)	6 (25.0%)	0.537
Normal-flux	184 (63.9%)	168 (63.6%)	16 (66.7%)	0.767
High-flux	16 (5.6%)	14 (5.3%)	2 (8.3%)	0.535
Tracheal intubation	8 (2.8%)	6 (2.3%)	2 (8.3%)	0.084
CPAP	32 (11.1%)	28 (10.6%)	4 (16.7%)	0.366
CRRT	5 (1.7%)	4 (1.5%)	1 (4.2%)	0.341
ECMO	4 (1.4%)	3 (1.1%)	1 (4.2%)	0.225
Glycemic control therapy				
Use of insulin	NA	NA	6 (25%)	NA
Oral glucose control agents	NA	NA	15 (62.5%)	NA
Combined	NA	NA	3 (12.5%)	NA
Outcomes				
Admission to ICU	27 (9.4%)	22 (8.3%)	5 (20.8%)	0.044
ARDS	3 (1.0%)	2 (0.8%)	1 (4.2%)	0.115
Clinical types^a^ (severe/critically severe)	30 (10.4%)	25 (9.5%)	5 (20.8%)	0.048

Data are presented as median (interquartile range) or number (percent). Abbreviations: NA: not applicable; CPAP: continuous positive airway pressure ventilation; CRRT: continuous renal replacement therapy; ECMO: extracorporeal membrane oxygenation; ICU: intensive care unit; ARDS: Acute Respiratory Distress Syndrome. ^a^Clinical types (severe/critically severe) were based on the notice on the issuance of a program for the diagnosis and treatment of novel coronavirus- (2019-nCoV-) infected pneumonia (7th edition) published by the General Office of National Health Committee.

**Table 3:** Risk factors of ICU admission for COVID-19 patients with DM.

	Univariable OR (95% CI)	*P* value	Multivariable OR (95% CI)	*P* value
Age (years)	1.09 (1.02-1.27)	<0.0001	1.07 (1.02-1.13)	0.007
Female sex (vs. male)	0.12 (0.01-1.27)	0.077	..	..
Hypertension	2.333 (0.216-25.245)	0.486		
CVD	2.33 (0.22-25.25)	0.486	..	..
Fever	0.75 (0.061-9.27)	0.823		
Cough	2.91 (0.27-31.21)	0.575	..	..
Expectoration	0.70 (0.06-7.85)	0.772	..	..
Fatigue	12.0 (0.81-77.43)	0.071	..	..
Myalgia	5.67 (0.56-57.23)	0.142	..	..
Headache	4.50 (0.23-88.24)	0.322	..	..
Respiratory rate ≥ 24 breaths per min	7.73 (3.22-38.56)	0.003	5.22 (2.26-16.58)	0.016
Lymphocyte count (×10^9^/L)			..	..
≤1.1	1.44 (0.19-11.04)	0.732		
>1.1	1 (ref)			
Blood glucose (mmol/L)	1.57 (0.64-7.25)	0.648	..	..
HbA1c (%)				
<7%	1 (ref)		1 (ref)	
≥7%	5.70 (2.19-15.46)	0.006	4.58 (1.82-10.55)	0.012
Platelet count (×10^9^/L)	1.00 (0.990-1.01)	0.996	..	..
Procalcitonin (ng/mL)			..	..
≤0.05	1 (ref)			
0.05-10	3.0 (0.248-36.33)	0.388		
>10	1.4 (0.07-28.12)	0.826		
CRP (mg/L)				
≤10	1 (ref)		..	..
>10	2.33 (0.22-25.25)	0.486	..	..
D-dimer (*μ*g/L)				
≤1000	1 (ref)		..	..
>1000	3.20 (0.29-34.58)	0.388	..	..
Myoglobin (*μ*g/L)	1.02 (0.99-1.01)	0.654	..	..
Creatine kinase (U/L)	1.01 (0.99-1.05)	0.664	..	..
ALT (U/L)				
≤50	1 (ref)		..	
>50	3.70 (1.53-8.83)	0.044	..	…
AST (U/L)				
≤40	1 (ref)		1 (ref)	..
>40	3.92 (1.82-9.62)	0.006	2.96 (1.58-8.85)	0.022

Abbreviations: OR: odds ratio; CI: confidence interval; CVD: cardiovascular disease; HbA1c: hemoglobin A1c; CRP: C-reactive protein; ALT: alanine transaminase; AST: aspartate aminotransferase.

**Table 4:** Risk factors of ICU admission for COVID-19 patients with non-DM.

	Univariable OR (95% CI)	*P* value	Multivariable OR (95% CI)	*P* value
Age (years)	1.07 (1.04-1.11)	<0.0001	1.05 (1.01-1.10)	0.006
Female sex (vs. male)	0.45 (0.28-1.12)	0.086	..	..
Hypertension	3.17 (1.31-7.69)	0.011		
CVD	3.98 (1.63-9.71)	0.009		
Fever	3.12 (0.90-10.88)	0.073		
Nausea or vomiting	2.75 (1.45-9.64)	0.016		
Diarrhea	7.78 (2.34-18.42)	0.003	4.62 (2.01-9.36)	0.022
Respiratory rate ≥ 24 breaths per min	7.66 (2.86-20.52)	<0.001	5.13 (1.18-16.82)	0.035
Lymphocyte count (×10^9^/L)			..	..
≤1.1	7.41 (2.78-19.78)	<0.0001	0.55 (0.19-2.75)	0.465
>1.1	1 (ref)		1 (ref)	
Procalcitonin (ng/mL)				
≤0.05	1 (ref)			
0.05-10	9.42 (1.17-15.92)	0.015		
>10	12.31 (2.32-24.52)	0.036		
CRP (mg/L)				
≤10	1 (ref)		1 (ref)	..
>10	6.87 (2.06-17.29)	<0.0001	5.19 (1.37-13.25)	0.009
BNP (ng/L)				
≤100	1 (ref)			
>100	12.05 (3.71-47.04)	0.003		
TNI (*μ*g/L)				
≤0.03	1 (ref)		1 (ref)	
>0.03	16.15 (4.90-32.16)	<0.0001	6.48 (1.17-21.38)	0.036
Myoglobin (*μ*g/L)			..	..
≤106	1 (ref)			
>106	8.22 (2.46-16.92)	0.017		
Creatine kinase (U/L)	1.004 (1.000-1.008)	0.082	..	..
≤310	1 (ref)			
>310	5.17 (1.42-13.58)	0.036		
ALT (U/L)				
≤50	1 (ref)		..	
>50	2.12 (0.66-6.79)	0.028	..	…
AST (U/L)				
≤40	1 (ref)		1 (ref)	..
>40	4.18 (2.12-13.19)	0.002	3.58 (0.85-19.24)	0.075

Abbreviations: OR: odds ratio; CI: confidence interval; CVD: cardiovascular disease; CRP: C-reactive protein; BNP: brain natriuretic peptide; TNI: troponin I; ALT: alanine transaminase; AST: aspartate aminotransferase.

## Data Availability

The data used to support the findings of this study are available from the corresponding author upon request.
